# When Social Skills Backfire: Social Media Overuse as a Pathway Linking Social Competence and Health Across Cultures

**DOI:** 10.3390/bs16060933

**Published:** 2026-06-05

**Authors:** Shaoyu Ye, Kevin K. W. Ho

**Affiliations:** 1 Institute of Library, Information and Media Science, University of Tsukuba, Tsukuba 305-8550, Japan; 2 Institute of Business Sciences, University of Tsukuba, Tokyo 112-0012, Japan; ho.kevin.ge@u.tsukuba.ac.jp

**Keywords:** social skills, self-presentation, social media use, Japan, Hong Kong, gastrointestinal problems, headache, respiratory infection, sleeping disturbance

## Abstract

This study investigates how self-presentation and social skills relate to physical health among university students in Japan and Hong Kong, with particular attention to the mediating roles of social media use and social support. We conducted two online surveys; as a result, 452 participants in Japan and 289 participants in Hong Kong were analyzed using multi-group structural equation modeling. Across both samples, social skills were directly associated with increased physical symptoms, and this direct association was substantially stronger than the indirect association through social support, which was linked to fewer physical symptoms. Notable cross-cultural differences also emerged. In Japan, self-presentation was directly associated with increased respiratory–sleep symptoms (R-SSs), and this direct association was stronger than the indirect pathway through posting frequency and time spent on social media. In contrast, in Hong Kong, no direct association between self-presentation and physical symptoms was detected, although an indirect association through social media usage time—leading to increased R-SS—emerged at a marginally significant level. These findings highlight the dual and context-dependent effects of personal attributes in digital environments, demonstrating that characteristics typically associated with positive social functioning may produce unintended health consequences online. By integrating personal factors, social media behaviors, and cultural context, this study advances understanding of digital well-being and physical health in contemporary societies.

## 1. Introduction

Previous studies have demonstrated that self-presentation on social media is associated with users’ physical health. For example, a meta-analysis by Zell et al. (2003) examined the relationships between self-enhancement, self-presentation and physical health outcomes—such as self-related health and biological markers—and found that these associations vary across different dimensions of physical health. Other research suggests that fitness-related self-presentation facilitates the receipt of social support, thereby enhancing confidence and motivation to engage in physical activity (H.-M. Kim, 2024). Recently, Segrin (2019) reported that deficits in social skills are associated with physical health problems among adults aged 18 to 91.

In addition, research on social media use and health has indicated that individuals with higher levels of social skills are more capable of forming supportive social networks through face-to-face (FTF) communication, which benefits physical health and is associated with lower loneliness (Ye & Ho, 2024b). Notably, in terms of users who combined X (formerly Twitter) and Instagram, personal factors, including social skills, were directly associated with increased perceived physical health, which could not be observed among users with other combinations of patterns.

Taken together, these findings suggest that personal factors—such as self-presentation and social skills—are associated with physical health both directly and indirectly through social support. These relationships may further vary according to patterns of social media use. Building on these implications, the present study examines how self-presentation and social skills are related to physical health among university students, with a particular focus on the mediating role of social support.

Furthermore, despite the growing number of studies on self-presentation, social skills, and social media use, few studies have integrated these factors to explain physical health outcomes across cultural contexts. Drawing on Hofstede’s cultural dimension theory (Hofstede, 2001), Japan is characterized by relatively higher levels of uncertainty avoidance than other Asian countries/regions, such as Hong Kong, one of the most internationalized cities in the Asia Pacific. Therefore, this study aims to investigate the similarities and differences in the relationships among these variables between Japan and Hong Kong.

Despite growing research on self-presentation, social skills, and social media use, few studies have examined how these factors jointly shape physical health outcomes across cultural contexts. Moreover, existing studies have rarely distinguished between different forms of social media engagement, such as posting frequency and time spent, or examined their sequential mediating roles. Addressing these gaps, the present study proposes and tests an integrated cross-cultural model linking self-presentation, social skills, social media use, social support, and physical health among university students in Japan and Hong Kong.

## 2. Literature Review

### 2.1. Self-Presentation and Physical Health

Prior research has demonstrated that self-presentation is associated with physical health; however, the findings have been mixed. Some studies have suggested that high levels of self-presentation are linked to behaviors that may harm physical health, such as malnutrition, substance use, and avoidance of seeking medical treatment (Leary et al., 1994). In addition, Ginis and Leary (2004) examined the two-component model of impression management—impression motivation and impression construction in the performance of health—damaging behaviors in physical activity and other contexts. They found that both features of the immediate situation (e.g., the number and identity of other people who are present, operating norms and roles) and characteristics of the individual (e.g., traits, values, goals, self-concept) affect the performance of health-damaging behaviors. These findings suggest that self-presentation can directly shape physical health outcomes through behavior choices. In addition, other studies have reported that self-presentation may also be negatively associated with mental health, as it can increase social physique anxiety (Driediger et al., 2016).

In contrast, other research suggests that self-presentation can function as a vicarious reinforcer that promotes engagement in physical exercise (Fox & Bailenson, 2009), thereby potentially improving physical health. For example, Liu et al. (2021) conducted a survey on 747 overweight college students in China and found that the higher scores in “attractiveness,” “weight control,” and “ability display,” the higher the psychological needs and exercise dependence; the higher the autonomy, competence and relationships, the lower the emotional, physiological and dynamic distress. These findings highlight the dual role of self-presentation, which may facilitate engagement in health-promoting behaviors while concurrently elevating the risk of maladaptive outcomes, such as compulsive exercise.

More recently, research has begun to examine online self-presentation and its health-related consequences. For instance, a systematic review by Bij de Vaate et al. (2020) found that self-presentation can either enhance or undermine well-being and body image, depending on its form (e.g., authentic vs. idealized). Given that body image is strongly related to eating behaviors, physical activities and somatic symptoms, these findings further suggest that self-presentation may be associated with physical health through the mediating role of body image.

### 2.2. Social Skills and Physical Health

Social skills, broadly defined as the ability to effectively perceive, interpret, and respond to social cues, are fundamental to interpersonal functioning and overall well-being. A growing body of research suggests that social skills play an important role in shaping physical health outcomes, albeit often indirectly. Compared with the mixed evidence regarding the relationships between self-presentation and physical health, findings on the relationship between social skills and physical health are relatively consistent. Prior research indicates that individuals with lower levels of social skills are more likely to experience social stress and loneliness, which can, in turn, adversely affect physical health (Segrin, 2019). Conversely, individuals with high levels of social skills—particularly those with higher socioeconomic status—are more likely to engage in healthier habits, such as maintaining balanced dietary habits (Furman et al., 2025).

In addition, Ye and Ho (2024b) found that university students with higher levels of social skills not only reported lower levels of loneliness and higher subjective well-being directly, but also demonstrated better physical health through the mediating role of social capital derived from FTF communication. Furthermore, Schüller et al. (2025) emphasized the importance of incorporating social skill development into health promotion programs.

On the other hand, emerging evidence suggests that the relationship between social skills and physical health may be bidirectional. For example, Misriandi and Susanto (2024) investigated the impact of physical activity on the social skills of 164 elementary school students in South Tangerang. They have found that regular participation in structured physical activities is significantly related to students’ social skills, with team-based sports showing the most substantial positive effects. Their findings suggest that physical activity can foster essential social skills, which in turn may contribute to students’ overall personal and academic development. Together, this research highlights a reciprocal relationship between social skills and physical health.

### 2.3. Social Media Use and Physical Health

With the increasing integration of social media and the Internet into daily life, a growing body of research has examined their relationships with physical health. Empirical studies suggested that social media use is associated not only with self-reported physical symptoms but also with physiological indicators such as inflammation. For instance, D. S. Lee et al. (2022) investigated how the amount of social media use relates to various indices of physical health among young adults who provided a blood sample and completed self-report surveys on social media use, somatic symptoms, and illness-related physician or health center visits, as well as whether they sought medical care for infection-related illnesses in the last 3 months. They found that social media use was positively associated with higher levels of C-reactive protein, more somatic symptoms, and more visits to the doctor or health centers for an illness. Their findings underscore the need for further research examining how different patterns of social media use relate to physical health, particularly in interaction with personal factors such as self-presentation and social skills.

In addition, Dibb (2019) has conducted a cross-sectional survey on 165 adults (mean age: 31.4 years, *SD* = 13.95, range 18–70) who used Facebook in the United Kingdom. The results showed that physical symptoms were associated with gender, Facebook use and positively interpreted upward comparison. Specifically, individuals who agreed more with the positively interpreted social comparison statements and who engaged more with Facebook perceived more physical symptoms. In Japan, Facebook use is relatively low among university students (6% in 2023), whereas platforms such as LINE, Instagram, X and Discord are more prevalent. (Ye & Ho, 2025). Moreover, individuals often use multiple platforms in combination, and these usage patterns are associated with different health outcomes (e.g., Ye & Ho, 2024a, 2024b, 2025). Notably, the combined use of Discord + X has been found to be most strongly associated with poorer perceived physical health (Ye & Ho, 2024b). These findings suggest that the relationship between social media use and physical health is context-dependent and may differ across cultural settings.

Furthermore, excessive social media use has been linked to a range of adverse health problems, including sleep disturbances (Viner et al., 2019), difficulties in weight control (Oduro et al., 2023), and reduced levels of physical activity (Ardesch et al., 2023).

The rapid global expansion of social media use among young people has heightened concerns about its potential adverse effects on physical health. Addressing this issue requires a more nuanced understanding of the mechanisms and contextual conditions linking social media use to physical health, as well as the development of effective risk-mitigation strategies. Prior studies have used overlapping terminology—such as passive use, excessive use, overuse, posting behavior, and usage time—sometimes interchangeably. To avoid conceptual ambiguity, the present study distinguishes between (a) posting frequency, which reflects active, goal-directed engagement, and (b) time spent, which more closely reflects passive, non-purposeful browsing. These two dimensions are theoretically and empirically separable and may have distinct implications for physical health. This distinction is consistent with Verduyn et al.’s (2017) differentiation between active and passive social media use.

### 2.4. Mediating Effects of Social Support

Social support has been widely recognized as a critical determinant of physical health. Extensive evidence from meta-analyses and epidemiological studies indicates that more social support is associated with better physical health outcomes, including lower risks of morbidity and mortality (Illinykh-Bair & Smith, 2026). For example, a meta-analysis synthesizing data from 300,000 participants found that stronger social relationships significantly increase the likelihood of survival (Holt-Lunstad et al., 2010), while another meta-analysis including more than 180 studies reported that social support is a significant predictor of various health outcomes, including physical symptoms and health-related behaviors (Wang et al., 2003). Moreover, social support has been linked to a range of physical health indicators, such as sleep quality, fatigue, and frailty (Lem et al., 2021). These effects are often explained through stress-buffering mechanisms—whereby social support reduces psychological stress and its physiological consequences—as well as through behavioral pathways that promote healthier lifestyles, such as increased physical activity (Uchino, 2006; Smith et al., 2017). Collectively, these findings suggest that social support plays both direct and indirect roles in shaping physical health.

In contemporary contexts, individuals increasingly rely on various social media platforms for different purposes, including receiving social support. However, the effectiveness of such support may depend on how social relationships are formed. In fact, Ye and Ho (2023) found that university students in Japan used X to receive social support, including emotional support during the first COVID-19 state of emergency; however, such support was not associated with their well-being. In addition, Ye and Ho (2024b) found that social networks formed through FTF communication were positively associated with better perceived physical health, whereas no comparable association was observed for social networks formed through online interactions. Furthermore, a three-level meta-analysis by L. Huang et al. (2026) reported a negative association between overall social support and problematic social media use, alongside a positive correlation between online social support and problematic social media use. These findings suggest that while more social support may be associated with less problematic social media use, reliance on online-derived support may, in some cases, be associated with increased problematic behaviors.

Given that passive social media use (e.g., browsing others’ posts without a specific purpose) has been shown to be negatively associated with subjective well-being, whereas active use (e.g., purposeful posting) is positively associated with subjective well-being (Verduyn et al., 2017), it is plausible that similar patterns may extend to physical health outcomes. Therefore, this study distinguishes between posting frequency and time spent on social media as separate dimensions of use, as posting frequency is conceptualized as an indicator of active, goal-directed engagement, whereas time spent is more likely to capture passive, non-purposeful use.

### 2.5. Cultural Differences

Culture plays a fundamental role in shaping individuals’ personality, cognition, and behavior. For example, Markus and Kitayama (1991) proposed that cultural contexts influence individuals’ self-construals, distinguishing between independent and interdependent selves. Specifically, individuals in more individualistic cultures tend to emphasize autonomy and personal goals, whereas those in more collectivistic cultures are more likely to prioritize social harmony and group-oriented behaviors.

In addition, research has shown that cultural background shapes individuals’ affective experiences and emotional responses in daily activities (J. L. Tsai, 2007). Such differences may extend to the use of social media, where individuals from different cultural contexts may experience and interpret the same online interactions in distinct ways. Consequently, even when using the same social media platforms, individuals from different cultural backgrounds may derive different emotional and psychological outcomes. These culturally shaped experiences may influence mental health indicators, such as their subjective well-being and depressive symptoms, and may further extend to physical health outcomes.

Culture has also been found to play an important role in the processes of receiving social support. For instance, H. S. Kim et al. (2008) demonstrated that the cultural background influences whether individuals seek social support, as well as how such support is interpreted. This suggests that when individuals use social media to obtain social support, cultural differences may shape both their patterns of interaction and the effectiveness of the support received.

Furthermore, a meta-analysis by C. Huang (2017) found that the relationship between social media use and well-being varies significantly across studies. Such inconsistencies may, in part, be attributable to cultural differences across samples, highlighting the potential role as a moderator in these relationships.

To sum up, these findings suggest that cultural context not only relates to individual behaviors and experiences but may also moderate the relationships among self-presentation, social skills, social support, social media use and physical health outcomes. Therefore, culture should be considered as an important factor in examining the relationships proposed in this study.

## 3. Research Model and Hypothesis Development

**Figure 1:**
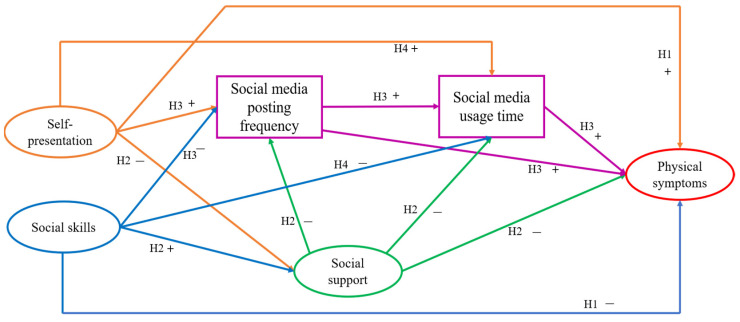
Research model in this study.

Based on the literature reviewed above, we developed the research model shown in Figure 1. Drawing on the cognitive bias model, previous studies have demonstrated that personal factors, such as the desire for self-presentation (Ye & Ho, 2024a, 2024b, 2025) and social skills (Ye & Ho, 2023, 2024b), are directly associated with mental health outcomes. It is therefore reasonable to extend this line of reasoning to physical health outcomes. In particular, self-presentation has been shown to be potentially detrimental to health, as individuals may engage in behaviors that prioritize impression management over well-being (Leary et al., 1994; Driediger et al., 2016). In contrast, social skills are generally associated with better health outcomes, as they facilitate effective interpersonal functioning, reduce stress, and promote access to social support (Segrin, 2019; Ye & Ho, 2024b; Furman et al., 2025).

Based on these findings, the present study proposes the following hypothesis:

**H1.**  *Self-presentation will be negatively associated with physical health directly, whereas social skills will be positively associated with physical health directly.*

Additionally, based on the social network mediation model, previous studies have indicated that lower levels of self- presentation and higher levels of social skills are able to form fruitful social networks which offer more social support, which in turn contributes to improved subjective well-being (Ye & Ho, 2023, 2024b, 2025) and reduced loneliness through face-to-face (FTF) communications (Igarashi, 2002). The social network mediation model proposes that personal dispositions influence mental and physical health indirectly through the formation and maintenance of social networks and has been widely applied in studies linking social skills, social capital, and health outcomes. Extending this framework to physical health, it is reasonable to expect that social support may also serve as an important mediating mechanism linking these personal factors to physical health outcomes.

Therefore, the present study proposes the following hypothesis:

**H2.**  *Social support mediates the relationships between self-presentation, social skills, and physical health, such that lower self-presentation and higher social skills are associated with more social support, which in turn is related to better physical health (fewer symptoms).*

Furthermore, as noted above, excessive social media use has been linked to a range of health problems (Viner et al., 2019; Ardesch et al., 2023; Oduro et al., 2023). It is therefore plausible that such negative effects will be more pronounced among individuals with higher levels of self-presentation and lower levels of social skills. Individuals who are highly concerned with self-presentation may engage more frequently in posting and impression management activities, which require increased time and effort on social media platforms. This heightened engagement may, in turn, contribute to poorer physical health outcomes. Therefore, this study proposes the following hypothesis:

**H3.**  *Social media overuse mediates the relationships between self-presentation, social skills, and physical health, such that higher self-presentation and lower social skills are associated with more social media overuse, which in turn is associated with poorer physical health (more symptoms).*

Although the relationship between posting frequency and time spent may be bidirectional, we model posting frequency as an antecedent to usage time because impression-management activities often require extended engagement (e.g., monitoring feedback, editing posts, responding to comments). This direction is consistent with prior work showing that self-presentation motives increase time spent on social media platforms (Fox & Bailenson, 2009). Nonetheless, we acknowledge the plausibility of the reverse direction and discuss this as a limitation.

Simultaneously, passive use of social media—such as spending extended periods browsing others’ posts without a specific purpose—has been found to be negatively associated with health outcomes (Verduyn et al., 2017). These negative effects may be particularly pronounced among individuals with higher levels of self-presentation and lower levels of social skills. Such individuals may be more likely to engage in passive consumption of social media content, which can increase social comparison and, therefore, contribute to adverse physical health outcomes. Therefore, this study proposes the following hypothesis:

**H4.**  *Passive social media use mediates the relationships between self-presentation, social skills, and physical health, such that higher self-presentation and lower social skills are associated with more passive use, which in turn predicts poorer physical health (more symptoms).*

Finally, drawing on Geert Hofstede’s cultural dimension theory (Hofstede, 2001), it has been noted that Japan is characterized by relatively higher levels of uncertainty avoidance than other Asian countries/regions, such as Hong Kong. Individuals in cultures with high uncertainty avoidance tend to be more sensitive to social evaluation and ambiguity, which may influence their patterns of social media use and interpersonal behavior. In addition, prior research has shown that a substantial proportion of Japanese individuals (Facebook users) report discomfort with engaging in self-presentations that involve constructing an idealized or inauthentic image of themselves on Facebook (Ye, 2019). This may partly explain why Japanese young people prefer social media platforms that afford a higher degree of visual anonymity, such as X.

Taken together, these cultural characteristics and preferences on social media use suggest that the relationships among self-presentation, social skills, social media use and physical health may differ between Japan and Hong Kong. Therefore, this study further examines the following hypothesis:

**H5.**  *Culture (Japan vs. Hong Kong) moderates the relationships proposed in H1–H4, such that the strength and/or direction of these paths differ between the two samples.*

## 4. Methodology

In order to examine the above hypotheses, we conducted online surveys in June 2024 with undergraduate students from four universities in the Kanto Region of Japan, and from late September 2024 to mid-May 2025 with two universities in Hong Kong. We successfully recruited 472 respondents, of whom 462 completed the survey in Japan and 458 completed the survey in Hong Kong. To ensure meaningful comparisons across cultures, only participants holding Japanese nationality (Japan sample) and Hong Kong SAR passports (Hong Kong sample) were included in the final analysis.

Before answering the survey, the participants were provided with written instructions and information, including the ethical approval process, purpose of the survey, research method, storage of materials, use of their data in other research, informed consent, voluntary participation and privilege to withdraw responses, privacy and protection of their personal information, publication of research results, and contact addresses. Those willing to participate completed a consent form before proceeding with the study. Data were analyzed using multi-group structural equation modeling (MGSEM). Measurement invariance testing indicated partial metric invariance, and non-invariant loadings were freely estimated across groups.

The implementation of the surveys was approved by the Research Ethics Review Committee in both Japan and Hong Kong. The questionnaire was administered in Japanese in Japan and English in Hong Kong. The survey was divided into three sections. Section A collected demographic data, including gender, age, nationality, mother language, grade, living styles (Table 1), and participants’ levels of desire for self-presentation and admiration (15 items from Kojima et al. (2003) and S. J. Lee et al. (1999), including “when I’m blamed for something, I make excuses,” “I make up excuses for poor performance,” and so on, measured from 5, very frequently, to 1, very infrequently), and social skills (18 items from Riggio (1986), such as “when I get depressed, I tend to bring down those around me,” “I have been told that I have ‘expressive’ eyes,” and so on, from 5, strongly agree, to 1, strongly disagree).

Section B collected data related to Internet usage time (from 7, more than 12 h, to 1, less than 2 h) by type of devices used (personal computers [PCs], mobile phones, and tablets), time spent, and purpose of social media use. In Japan, the social media included LINE, X, Instagram, Discord, and TikTok, while in Hong Kong, it included WhatsApp, WeChat, Instagram, X, TikTok, and so on. We also measured their frequency of posting (from 5, almost every day, to 1, seldom) on each social media platform and post content type, and the percentages of people whom they followed (friends/acquaintances, lovers, family/relatives, online-only acquaintances/friends, others).

Section C measured participants’ social support received from others (12 items from Zimet et al. (1988), such as “there is a special person who is around when I am in need,” “there is a special person with whom I can share my joys and sorrows,” and so on, measured with 7. strongly agree, 4. neutral, and 1, strongly disagree), and physical health (14 items from Schat et al. (2005); Items 1–11 with frequency from 7. not at all, to 1. all the time [such as “how often have you had difficulty getting to sleep at night?” “how often have you experienced headaches?” and so on]; Items 12 and 13 with “the number of times” from 7. 0 times, to 1. 7 times [“how many times have you had minor colds?” and “how many times have you had respiratory infections more severe than minor colds that ‘laid you low’?”]; and Item 14 [when you had a bad cold or flu, how long did it typically last?] with “the number of days,” from 1. 1 day, to 7. 7 days)^1^.

## 5. Results

### 5.1. Information of Analyzed Participants

To ensure meaningful cross-cultural comparison, this study focused exclusively on local residents in each sample. The Japan sample was restricted to university students holding Japanese nationality (n = 452), and the Hong Kong sample was limited to university students holding Hong Kong SAR passports (n = 289). For clarity, these groups are henceforth referred to as Japanese university students and Hong Kong university students, respectively. Table 1 summarizes the demographic information and social media use of the analyzed participants in Japan and Hong Kong. In both samples, female participants outnumbered male participants (43.4% in Japan and 64.4% in Hong Kong). Regarding academic standing, participants from Japan were relatively evenly distributed across all four undergraduate years, whereas more than half of the participants from Hong Kong were first-year students. Regarding residency status, over 60% of participants in Japan were living alone, whereas in Hong Kong, 73.4% lived with their families and 17.0% lived in shared accommodations.

Regarding social media use, we calculated the top five most frequently used, with at least 5% of the usage time among the total time spent on all social media in both samples. As a result, in Japan, the five most frequently used platforms were LINE (99.8%), X (88.1%), Instagram (80.3%), Discord (42.3%) and TikTok (24.6%). In Hong Kong, the corresponding platforms were WhatsApp (98.3%), Instagram (98.3%), WeChat (40.8%), X (24.9%) and TikTok (14.9%). In terms of posting content, as Table 2 indicates, participants in Japan most frequently used X to share common interests and hobbies, while Instagram was primarily used to present an idealized lifestyle and to share photos and videos. LINE and Discord were mainly used to reply to their friends to maintain daily connections with friends. In contrast, participants in Hong Kong primarily used Instagram across multiple functions, including sharing common hobbies, presenting their lives, posting photos and videos, and communicating with friends. Platforms such as WhatsApp and WeChat were mainly used for daily communication.

Regarding average monthly Internet use, participants from Hong Kong spent more time online than those from Japan across all three types of devices (hours per month). In contrast, the average posting frequency across all social media platforms was initially comparable between the two groups. To further examine these differences, we conducted an independent *t*-test to compare the time spent on different devices, total time spent on all social media, and overall posting frequency. The results are summarized in Table 3. As shown in Table 3, participants in Hong Kong reported significantly longer time spent on computers, smartphones, tablets and social media overall compared to participants in Japan. In addition, posting frequency across all social media platforms was also significantly higher among participants in Hong Kong than in Japan.

### 5.2. Factor Analyses and Internal Reliability

In this study, we combined responses from both Japanese students and Hong Kong students in the same data set and conducted factor analyses for self-presentation, social skills, social support and physical health using the same methods as in previous studies. The results are summarized in Table 4.

For self-presentation, three subscales were identified, all of which demonstrated high internal reliability. For social skills, five subscales were initially identified; however, only four factors demonstrated acceptable internal reliability and were used in the further analysis. Regarding social support, three factors with high internal reliability were identified in the previous study. For physical health, four factors were identified, consistent with the structure reported in previous research. As shown in Table 4, the fourth factor (sleep disturbance) exhibited comparatively low internal reliability (0.55). However, although 0.5–0.6 for Cronbach’s Alpha is not ideal but acceptable (Peterson, 1994), the structure is the same as the previous study’s, which indicates that it does not necessarily imply poor construct reliability, especially in SEM (Sijtsma & Pfadt, 2021); therefore, we included it for further analysis in this study.

As stated earlier, following the previous study, higher scores were interpreted as indicating more physical symptoms. Accordingly, in the present study, “physical health” refers to more physical symptoms.

Additionally, independent *t*-tests were conducted to compare these factors across the two samples; the results are summarized in Table 4. As shown in Table 4, participants in Japan reported significantly higher levels of “excuse” in self-presentation and more social support from family. In contrast, participants in Hong Kong reported significantly higher levels of the four factors in social skills, except for “social control.” Moreover, participants in Hong Kong reported significantly higher levels of physical symptoms, including “gastrointestinal problems (GP)”, “headaches,” and “respiratory infection (RI).”

### 5.3. Results of SEM Analyses

Data from both samples were analyzed using SmartPLS 4.1.1.5 (Ringle et al., 2024). We initially estimated a model that included all hypothesized paths and then sequentially removed nonsignificant paths in order of decreasing statistical significance (i.e., largest *p*-value), similar to a backward elimination procedure, until all remaining paths met the criterion of *p* < 0.10. Paths with *p*-values between 0.05 and 0.10 were treated as trend level effects and interpreted cautiously, consistent with exploratory SEM practices. In addition, we conducted MGSEM to investigate the similarities and differences between the two samples. Furthermore, previous studies have indicated that GP and headaches are often linked via “pain” or “neuro-visceral” cluster (C. H. Tsai, 2010), which forms a coherent neuro-gastrointestinal cluster (J. H. Kim et al., 2023), while RI and “sleeping disturbance (SD)” are symptoms commonly grouped with the systemic/cardiopulmonary-regulation cluster (Kalantari et al., 2022). Therefore, we divided physical symptoms into two groups in the SEM accordingly, namely, “GI-Neurological symptoms (GI-NS)” refers to GP and headaches, while “Respiratory-Sleep symptoms (R-SS)” refers to RI and SD.

During the cleaning process, “exemplification” under self-presentation and “social expressivity” under social skills of the model for Japan, and “emotional control” under social skills of the model for Hong Kong, were removed due to low factor loading and high *p*-value. The model fit indices are shown in Table 5. Based on the previous studies (e.g., Hair et al., 2022; Henseler et al., 2016), a threshold of <0.10 is considered an acceptable heuristic for exploratory, predictive PLS-SEM contexts (Hair et al., 2019). Therefore, the Japan sample satisfies the threshold, while the Hong Kong sample sits slightly above it. Recent methodological studies suggest that SRMR in PLS-SEM can be more lenient in complex models, multi-group cross-cultural comparisons, and models mapping developing theoretical frameworks. However, other global fit indices, i.e., d_ULS, d_G, and NFI, are not optimal. This reflects the exploratory, cross-cultural, and complex nature of the proposed model, and results should therefore be interpreted with appropriate caution. Additionally, the global goodness-of-fit is not the primary evaluation criterion in PLS (variance-based) SEM. Accordingly, model evaluation in this study focuses primarily on path coefficients, explained variance (R^2^), and predictive relevance.

The analyzed results are summarized in Table 6. Several similarities were observed across the two samples. In both samples, self-presentation was significantly associated with higher posting frequency, which led to longer time spent on social media that was associated with more R-SSs. In addition, social skills were positively associated with more social support, which was significantly associated with fewer physical symptoms (both GI-NSs and R-SSs). Furthermore, self-presentation was associated with social media usage time (with a marginally significant relationship in Japan, with *p* = 0.06, and a significant relationship in Hong Kong), and social skills were positively associated with more R-SSs (with a significant relationship in Japan and a marginally significant relationship, with *p* = 0.06, in Hong Kong).

**Figure 2:**
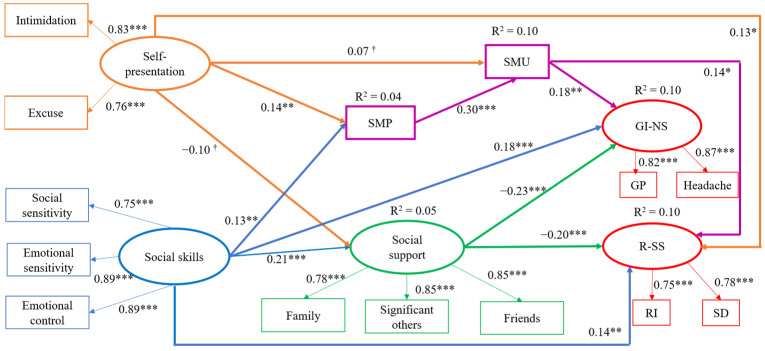
Analyzed results for the Japan sample. *Notes*: *** *p* < 0.001; ** *p* < 0.01; * *p* < 0.05; ^†^ *p* < 0.10.

**Figure 3:**
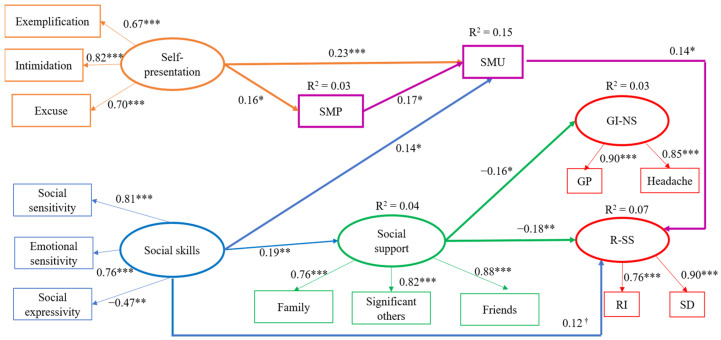
Analyzed results for the Hong Kong sample. *Notes*: *** *p* < 0.001; ** *p* < 0.01; * *p* < 0.05; ^†^ *p* < 0.10.

Notable differences were also identified between the two samples. In the Japanese sample, social skills were significantly associated with higher posting frequency and more GI-NSs, and their time spent on social media was also associated with more GI-NSs. In addition, self-presentation was also significantly associated with more R-SSs, and their self-presentation was marginally negatively associated with less social support (*p* = 0.07). In contrast, in the Hong Kong sample, social skills were significantly associated with longer time spent on social media. The results for each sample are illustrated in Figure 2 and Figure 3.

Mediating analyses were conducted to examine the distinct patterns across the two samples (see Table 7). The results revealed a consistent “protective pathway” in both Japan and Hong Kong, whereby social support significantly mediated the relationship between social skills and physical symptoms. Specifically, higher levels of social skills were associated with fewer GI-NSs (Japan: β = −0.05, *p* < 0.01; Hong Kong: β = −0.03, *p* = 0.07) and R-SSs (Japan: β = −0.04, *p* < 0.01; Hong Kong: β = −0.03, *p* < 0.05) through increased social support. In contrast, social support did not significantly mediate the association between self-presentation and physical symptoms in either sample.

Additional indirect pathways involving social media behaviors showed more complex and context-specific patterns. In Hong Kong, self-presentation was marginally associated with higher R-SSs through increased social media usage time (β = 0.03, *p* = 0.06), and social skills were also marginally positively linked to R-SSs via social media usage time (β = 0.02, *p* = 0.06). Posting frequency was further marginally positively associated with R-SSs through social media usage time in Hong Kong (β = 0.02, *p* = 0.08).

In the Japan sample, several indirect effects involving posting frequency and social media usage time were significant. Posting frequency was positively associated with both GI-NSs (β = 0.05, *p* < 0.01) and R-SSs (β = 0.04, *p* < 0.05) via social media usage time. In addition, small but marginally significant indirect effects were observed for self-presentation through posting frequency and social media usage time on both GI-NSs and R-SSs (both βs = 0.01, *p* = 0.06 and 0.09, respectively), as well as for social skills through posting frequency and social media usage time on GI-NSs (β = 0.01, *p* = 0.09).

Overall, these findings suggest that social support consistently plays a protective role in reducing physical symptoms across both cultural contexts. However, pathways involving social media use—particularly increased usage time and posting frequency—may represent potential risk mechanisms associated with more symptoms.

## 6. Discussion

### 6.1. General Discussion

This study examined how self-presentation and social skills relate to physical health among university students in Japan and Hong Kong, with particular attention to the mediating roles of social media use and social support. By integrating personal dispositions, online behaviors, and cultural contexts, the findings revealed both shared mechanisms and culturally specific differences. Beyond identifying cultural variation, this study tested four hypotheses (H1–H4) regarding direct and indirect relationships among key variables, while also considering cultural differences between Japan and Hong Kong (H5).

First, the results demonstrated culturally distinct direct associations. In Japan, both self-presentation and social skills were directly and significantly associated with increased respiratory–sleep symptoms (R-SSs), and social skills were also directly associated with increased gastrointestinal–neurological symptoms (GI-NSs). In contrast, in Hong Kong, only social skills showed a marginally significant association with more R-SSs. These findings suggest that both self-presentation and social skills may exert stronger detrimental effects on physical symptoms (both GI-NSs and R-SSs) in Japan than in Hong Kong. Several reasons may help explain these differences. As shown in Figure 2 and Figure 3, the structure of self-presentation differed across samples: the Japan sample included “intimidation” and “excuse”, whereas the Hong Kong sample additionally exhibited “exemplification”. Moreover, although social skills comprised the same three factors in both samples, the effect of “social expressivity” diverged sharply, with a negative effect in Hong Kong but a strongly positive effect in Japan. These structural and functional differences likely contribute to the distinct patterns of association observed across the two cultural contexts. Accordingly, H1 was supported for the first half component in Japan, while the second half component was in the opposite direction in both samples.

In addition, in both Japan and Hong Kong, social skills were significantly and positively associated with increased social support, whereas self-presentation showed a marginally significant negative association with social support in Japan. Social support, in turn, was negatively associated with physical symptoms (both GI-NSs and R-SSs), underscoring its protective role in physical health. These findings are consistent with prior research highlighting the beneficial effects of social support on well-being and loneliness (e.g., Ye & Ho, 2024b, 2025). Accordingly, H2 was fully supported in Japan and partially supported in Hong Kong, highlighting the importance of fostering social support in digital contexts. Although no evidence of curvilinear relationships was found in the present data, future research should examine potential nonlinear effects of social support.

Furthermore, this study proposed that individuals with higher levels of self-presentation may be more likely to overuse social media and are thereby associated with increased physical symptoms. As shown in Table 7, in Japan, self-presentation was indirectly associated with both GI-NSs and R-SSs through increased posting frequency and longer time spent on social media, although these relationships were marginally significant. No comparable pathways could be observed in Hong Kong. Instead, in Hong Kong, self-presentation was indirectly associated with R-SSs through increased social media usage time at a marginally significant level. These results suggest that while the mechanisms linking self-presentation to physical symptoms differ across cultures, social media overuse remains a relevant pathway.

Importantly, the structure of self-presentation differed across the two samples. In Japan, it comprised the subscales of “intimidation” and “excuse”, whereas in Hong Kong it included “intimidation,” “excuse,” and “exemplification. As shown in Table 4, Japanese participants scored significantly higher on “excuse,” while no difference was observed for “intimidation.” This pattern can be interpreted through cultural differences in social ecology and impression management. In Japan, low relational mobility makes interpersonal relationships stable and difficult to replace, increasing concerns about reputation and social evaluation (Hofstede, 2001; Kusakabe et al., 2024). Under such conditions, “excuse” serves as a defensive self-presentation strategy that mitigates blame and protects one’s social image. Individuals are therefore more likely to adopt excuse-based strategies to avoid social exclusion. This tendency is further reinforced by cultural norms emphasizing self-effacement and modesty (Yamagishi et al., 2012). In contrast, Hong Kong—more strongly influenced by Western cultural values—encourages a more balanced and flexible self-presentation style, placing less emphasis on defensive strategies. These findings also align with cultural differences in independent versus interdependent self-construals, which shape how individuals interpret social evaluation and social media interactions.

These cultural tendencies also interact with patterns of social media use. Participants in Japan preferred platforms with higher levels of visual anonymity (e.g., X and Discord), which aligns with their more indirect or defensive self-presentation styles. In contrast, participants in Hong Kong more frequently used platforms with lower visual anonymity (e.g., WhatsApp, Instagram, and WeChat) to maintain interpersonal relationships while simultaneously engaging in self-presentation. Notably, although participants in Hong Kong reported both higher posting frequency and longer time spent on social media, usage time was only associated with R-SSs but not with GI-NSs. This pattern is consistent with previous research suggesting that the impact of media overuse—including social media—on different physical symptoms depends not only on duration but also on usage patterns and cultural context (Jackson & Wang, 2013; I. G. Lee & Son, 2025).

Regarding social skills, a more complex pattern emerged in both samples. Social skills were associated not only with increased social support (protective pathway) but also with higher posting frequency and longer time spent on social media (risk pathway). In Japan, this risk pathway was marginally associated with increased GI-NSs, while in Hong Kong, social skills were indirectly associated with R-SSs through longer usage time spent on social media. These findings suggest a dual role of social skills in digital environments. Individuals with higher social skills—such as social sensitivity, emotional sensitivity, and emotional control—may be more attuned to social cues and motivated to engage actively in online interactions. Although these traits are advantageous in offline contexts, they may heighten pressure for responsiveness and impression management online, leading to excessive engagement and potential health risks. Cultural context may further intensify these dynamics. In Japan, higher uncertainty avoidance may amplify sensitivity to social evaluation, encouraging feedback-seeking and self-monitoring behaviors that prolong online engagement. Furthermore, platform differences may also contribute. Japanese participants more frequently used platforms such as X and Discord, which afford visually anonymous and interest-based interactions, potentially fostering unbounded engagement. For individuals with higher social skills, such environments may increase susceptibility to social media fatigue, thereby elevating risks for physical symptoms.

Overall, H3 and H4 were partially supported for self-presentation in both samples, whereas social skills were opposite to those hypothesized. The cultural differences observed between Japan and Hong Kong further supported H5. Taken together, these findings underscore the importance of considering cultural context when examining the health implications of social media use. In addition, the direct associations between self-presentation/social skills and physical symptoms in Japan were substantially stronger than the indirect associations with physical symptoms through social media overuse, and the direct association between social skills and R-SSs was also stronger in Hong Kong. These patterns suggest that personal dispositions may exert a more immediate influence on physical health than online behaviors alone. Interventions aimed at reducing excessive self-presentation and promoting balanced, intentional social media engagement—particularly among individuals with higher social skills—may help mitigate negative health outcomes. Given the exploratory and cross-sectional nature of this study, however, the findings should be interpreted as preliminary evidence rather than definitive causal pathways.

### 6.2. Practical Implications

The findings offer several practical implications for universities, health practitioners, and policymakers seeking to promote healthier digital engagement among young adults.

First, the results highlight the need to address excessive self-presentation, particularly in Japan, where self-presentation showed strong direct associations with respiratory–sleep symptoms. Interventions that encourage more authentic communication and reduce pressure for impression management—such as digital literacy programs, workshops on online identity, or campus-wide campaigns promoting realistic self-expression—may help mitigate these risks.

Second, the study reveals a dual role of social skills: while social skills were associated with increased social support, they were also associated with increased posting frequency and longer time spent on social media, which were in turn linked to increased physical symptoms. This suggests that students with higher social skills may require guidance on boundary-setting, self-regulation, and mindful responsiveness in digital environments. Programs that teach students how to manage social expectations online, avoid over-engagement, and recognize early signs of social media fatigue may be particularly beneficial.

Third, the protective role of social support across both cultural contexts underscores the importance of strengthening offline social networks. Universities can promote peer-support systems, mentoring programs, and community-building activities that reduce reliance on online interactions for emotional needs. Given that online-derived support may not always translate into improved well-being, especially in Japan, fostering face-to-face social capital remains essential.

Finally, the cultural differences observed in this study suggest that interventions should be culturally tailored. In Japan, where uncertainty avoidance is higher and visually anonymous platforms are more common, strategies should focus on reducing evaluation anxiety and promoting healthier engagement with anonymous or interest-based platforms. In Hong Kong, where students use more identity-based platforms and engage more frequently in posting, interventions may focus on managing impression motives and reducing social comparison.

### 6.3. Limitations and Future Directions

Several limitations warrant consideration. First, the cross-sectional design precludes causal inference. Longitudinal or experimental studies are needed to clarify temporal ordering among variables. Second, reliance on self-report measures may introduce bias; future research should incorporate objective digital trace data or physiological indicators. Third, although global model fit indices were reported, the SRMR value for the Hong Kong sample slightly exceeded commonly suggested thresholds, which may reflect model complexity, cross-cultural heterogeneity, and the exploratory nature of the proposed model. In line with recent PLS-SEM guidelines, model evaluation in this study therefore relied primarily on path coefficients, explained variance, and predictive relevance rather than strict global fit criteria. Fourth, differences in factor structures across the two cultural groups suggest the need for more rigorous assessment of measurement invariance in future studies. Finally, the sample consisted exclusively of university students, which may limit the generalizability of the findings to other populations.

## 7. Conclusions

This study provides new insights into the complex relationships among personal dispositions, social media use, social support, and physical health among university students in Japan and Hong Kong. Across both contexts, self-presentation emerged as a consistent risk factor for poorer physical health, operating either directly or indirectly through increased social media engagement, while social skills demonstrated a dual role: promoting social support yet also increasing susceptibility to excessive social media engagement. Importantly, the pathways linking these factors to physical symptoms differed across cultural contexts. Social skills contributed to both protective and risk pathways in both samples, with stronger risk pathways than the protective ones. Additionally, in Japan, self-presentation also played a central role in predicting increased symptoms (both GI-NSs and R-SSs). These findings highlight that traits typically associated with positive social functioning may produce unintended health consequences when expressed in digital environments and that such effects are shaped by cultural norms surrounding social evaluation and communication.

Overall, this study underscores that digital well-being is not solely a function of individual behavior but emerges from the dynamic interplay between personal attributes, social environments, and cultural context. By distinguishing between posting frequency and usage time on social media and examining their sequential roles, this research provides a more nuanced understanding of how social media use is related to physical health and offers a foundation for culturally sensitive interventions aimed at promoting healthier digital engagement.

## Figures and Tables

**Table 1:** Participants’ information in Japan and Hong Kong.

Demographics	Japan	Hong Kong
Gender		
Males	43.4%	35.6%
Females	54.9%	64.4%
Others	1.8%	0.0%
Average age (in years)	20.0 (SD: 1.28)	19.2 (SD: 1.46)
Grade		
First year	21.0%	62.6%
Second year	24.1%	18.7%
Third year	28.5%	12.8%
Fourth and higher years	26.3%	5.9%
Residency status		
Alone	61.9%	5.9%
With family	33.2%	73.4%
With friends	3.8%	3.8%
Room share	1.1%	17.0%
Social media		
LINE	99.8%	
X (Twitter)	88.1%	24.9%
Instagram	80.3%	98.3%
Discord	42.3%	
TikTok	24.6%	14.9%
WhatsApp		98.3%
WeChat		40.8%

**Table 2:** Participants’ post content on each social media platform in Japan and Hong Kong.

	LINE	X (Twitter)	Instagram	Discord	TikTok	WhatsApp	WeChat
Common hobby (%)	28.8	47.0	29.5	31.4	13.5		
		15.3	47.9		18.6	12.7	
Fulfilling lives (friends/selves) (%)			45.6		2.7		
		9.7	53.9		23.3		22.9
Photos, videos, etc. (%)	35.9	35.4	60.3	22.0	9.0		
		9.7	77.1		30.2	41.2	39.0
Replies to friends (%)	54.6	32.7		57.1	2.7		
			69.0		23.3	81.7	58.5
Daily friendship (%)	39.9		24.0	21.5			
			68.0			57.8	41.5
Reports and grades (%)							
						16.2	
Masochistic (%)		24.6					
						
Do not post (%)	19.3	36.7	25.3	19.4	77.5		
		84.7			70.8		30.5

*Notes*: We report the post content among the top five most frequently used social media in each sample. The percentages in Japan are in the first row of each group, and the percentages in Hong Kong are in the second row.

**Table 3:** Results for the *t*-test among common variables.

Items	Japan	Hong Kong	*t*-Statistics
Internet and social media use ^1^			
Internet usage time via PC	105.4	130.9	3.94 ***
Internet usage time via smartphone	168.5	202.6	5.46 ***
Internet usage time via Tablet	31.5	90.2	10.79 ***
Total usage time on social media	170.3	220.7	4.31 ***
Total social media posting frequency	49.7	47.9	−0.90

*Notes*: *** *p* < 0.001. Time spent on Internet and social media was counted monthly. ^1^ In our statistical analysis, usage time was converted to months as follows: 0–2 h were “30,” 2–4 h were “90,” 4–6 h were “150,” 6–8 h were “210,” 8–10 h were “270,” 10–12 h were “330,” and 12 h or more were “360.” In addition, as in previous studies, the posting frequency was “0” for “not use,” “seldom” as “0.5”, “one per month” as “1,” “once per week” as “5,” “several times a week” as “15,” and “almost every day” as “30.”

**Table 4:** Detailed information for the scales in Japan and Hong Kong.

Scales	Factors	Japan	Hong Kong	*t*-Test
Self-presentation	Exemplification (α = 0.84; 17.78%)	3.13	3.25	1.83 ^†^
	Intimidation (α = 0.84, 17.75%)	2.18	2.24	0.88
	Excuse (α = 0.82; 15.87%)	3.46	3.18	4.50 ***
Socal skills	Social sensitivity (α = 0.78; 12.27%)	2.75	2.88	2.06 *
	Emotional sensitivity (α = 0.68; 8.54%)	2.79	3.26	7.56 ***
	Emotional control (α = 0.66; 7.47%)	3.07	3.42	5.17 ***
	Social expressivity (α = 0.55; 6.32%)	2.52	2.74	3.52 ***
	Social control (α = 0.50; 6.17%)	3.67	3.57	1.76 ^†^
Social support	Family (α = 0.90; 24.47%)	5.66	5.01	6.30 ***
	Significant others (α = 0.91; 23.95%)	5.63	5.39	2.38 *
	Friends (α = 0.90; 23.63%)	5.52	5.55	0.33
Physical symptoms	Gastrointestinal problems (GP) (α = 0.80; 15.03%)	1.89	2.33	5.00 ***
	Headaches (α = 0.81; 12.45%)	1.86	2.40	6.01 ***
	Respiratory infection (RI) (α = 0.61; 9.79%)	1.77	2.30	6.37 ***
	Sleeping disturbance (SD) (α = 0.55; 8.82%)	2.47	2.56	1.19

*Notes*: *** *p* < 0.001; * *p* < 0.05; ^†^ *p* < 0.10.

**Table 5:** Model fit indices of PLS-SEM.

Index	Japan (n = 452)	Hong Kong (289)
SRMR	0.099	0.119
d_ULS	1.038	1.695
d_G	0.330	0.396
NFI	0.412	0.317

*Notes*: SRMR = Standardized Root Mean Square Residual; d_ULS = Unweighted Least Squares Discrepancy; d_G = Geodesic Discrepancy; NFI = Normed Fit Index.

**Table 6:** Path coefficients for two groups.

Path	Japan	Hong Kong
Social media usage time (SMU)	R^2^ = 0.10	R^2^ = 0.15
Self-presentation → SMU	0.07 ^†^	0.23 ***
SMP → SMU	0.30 ***	0.17 *
Social skills → SMU	**–**	0.14 *
Social media posting frequency (SMP)	R^2^ = 0.04	R^2^ = 0.03
Self-presentation → SMP	0.14 **	0.16 *
Social skills → SMP	0.13 **	–
Social support	R^2^ = 0.05	R^2^ = 0.04
Self-presentation → Social support	−0.10 ^†^	–
Social skills → Social support	0.21 ***	0.19 **
GI-Neurological symptoms [GI-NS]	R^2^ = 0.10	R^2^ = 0.03
SMU → GI-NS	0.18 **	–
Social skills → GI-NS	0.18 ***	–
Social support → GI-NS	−0.23 ***	−0.16 *
Respiratory-Sleep symptoms [R-SS]	R^2^ = 0.10	R^2^ = 0.07
Self-presentation → R-SS	0.13 *	–
SMU → R-SS	0.14 *	0.14 *
Social skills → R-SS	0.14 **	0.12 ^†^
Social support → R-SS	−0.20 ***	−0.18 **

*Notes*: *** *p* < 0.001; ** *p* < 0.01; * *p* < 0.05; ^†^ *p* < 0.10.

**Table 7:** Indirect (mediation) effects of the structured model related to physical symptoms.

Indirect Paths	Japan	Hong Kong
Effects on physical symptoms:		
Self-presentation → SMU → GI-NS	n.s.	
Self-presentation → SMU → R-SS	n.s.	0.03 ^†^
Social skills → SMU → R-SS		0.02 ^†^
Social skills → SMP → SMU → GI-NS	0.01 ^†^	
Social skills → SMP → SMU → R-SS	n.s.	
Self-presentation → SMP → SMU		n.s.
Self-presentation → SMP → SMU → GI-NS	0.01 ^†^	
Self-presentation → SMP → SMU → R-SS	0.01 ^†^	n.s.
Self-presentation → Social support → GI-NS	n.s.	
Self-presentation → Social support → R-SS	n.s.	
SMP → SMU → GI-NS	0.05 **	
SMP → SMU → R-SS	0.04 *	0.02 ^†^
Social skills → Social support → GI-NS	−0.05 **	−0.03 ^†^
Social skills → Social support → R-SS	−0.04 **	−0.03 *

*Notes*: ** *p* < 0.01; * *p* < 0.05; ^†^ *p* < 0.10. n.s. = not significant. Bootstrap sample size = 5000.

## Data Availability

The dataset is available upon reasonable request from the corresponding author.

## Note

1 When calculating the physical health scores, higher scores were considered to indicate more physical symptoms. Therefore, in this study, physical health refers to physical symptoms.
